# Adaptation Duration Dissociates Category-, Image-, and Person-Specific Processes on Face-Evoked Event-Related Potentials

**DOI:** 10.3389/fpsyg.2015.01945

**Published:** 2015-12-22

**Authors:** Márta Zimmer, Adriana Zbanţ, Kornél Németh, Gyula Kovács

**Affiliations:** ^1^Department of Cognitive Science, Budapest University of Technology and EconomicsBudapest, Hungary; ^2^Faculty of Philosophy and Education, University of ViennaVienna, Austria; ^3^Department of Cognitive Neuroscience, Institute of Psychology, Friedrich Schiller University JenaJena, Germany; ^4^DFG Research Unit Person Perception, Friedrich Schiller University JenaJena, Germany

**Keywords:** neural adaptation, adaptation duration, face processing, event-related potentials, face after-effect

## Abstract

Several studies demonstrated that face perception is biased by the prior presentation of another face, a phenomenon termed as face-related after-effect (FAE). FAE is linked to a neural signal-reduction at occipito-temporal areas and it can be observed in the amplitude modulation of the early event-related potential (ERP) components. Recently, macaque single-cell recording studies suggested that manipulating the duration of the adaptor makes the selective adaptation of different visual motion processing steps possible. To date, however, only a few studies tested the effects of adaptor duration on the electrophysiological correlates of human face processing directly. The goal of the current study was to test the effect of adaptor duration on the image-, identity-, and generic category-specific face processing steps. To this end, in a two-alternative forced choice familiarity decision task we used five adaptor durations (ranging from 200–5000 ms) and four adaptor categories: adaptor and test were identical images—Repetition Suppression (RS); adaptor and test were different images of the Same Identity (SameID); adaptor and test images depicted Different Identities (DiffID); the adaptor was a Fourier phase-randomized image (No). Behaviorally, a strong priming effect was observed in both accuracy and response times for RS compared with both DiffID and No. The electrophysiological results suggest that rapid adaptation leads to a category-specific modulation of P100, N170, and N250. In addition, both identity and image-specific processes affected the N250 component during rapid adaptation. On the other hand, prolonged (5000 ms) adaptation enhanced, and extended category-specific adaptation processes over all tested ERP components. Additionally, prolonged adaptation led to the emergence of image-, and identity-specific modulations on the N170 and P2 components as well. In other words, there was a clear dissociation among category, identity-, and image-specific processing steps in the case of longer (3500 and 5000 ms) but not for shorter durations (< 3500 ms), reflected in the gradual reduction of N170 and enhancement of P2 in the No, DiffID, SameID, and RS conditions. Our findings imply that by manipulating adaptation duration one can dissociate the various steps of human face processing, reflected in the ERP response.

## Introduction

The ability of the visual system to rapidly adjust to changing environmental conditions is one of its key characteristics (Mesik et al., 2013). Following the prolonged exposure of a visual stimulus, a subsequently viewed stimulus sometimes appears conspicuously distorted. This phenomenon, termed as adaptation-related after-effect, can affect the perception of various stimulus attributes (Müller et al., 2009). Moreover, adaptation has served as a powerful psychophysical tool in the past for demonstrating selective neural sensitivities to different stimulus dimensions from low-level stimulus features (such as contrast, orientation, spatial frequency, texture, or motion) to high-level object and face properties (Fang et al., 2006). Regarding faces, several studies have demonstrated that the way we perceive human faces can be systematically biased by a previously presented face. At the behavioral level, such face-adaptation-related after-effects are present for various dimensions or aspects of face processing—such as identity (Leopold et al., 2001), distortion (Webster and MacLin, 1999), race, expression, gender (Webster et al., 2004; Kovács et al., 2005, 2006, 2007), attractiveness (Rhodes et al., 2003), or eye-gaze direction (Schweinberger et al., 2007a). It is worth noting, however, that in some cases, repeated presentations of a given stimulus (or subsequent presentations of two stimuli from the same category) do not lead to perceptual biases (as in the case of adaptation-related after-effects) but rather to behavioral facilitations. The latter phenomenon is usually referred to as priming, typically associated with faster and/or more accurate responses. Although these two phenomena share some properties, such as their size-, viewpoint-, or position-invariances, they are clearly distinguishable regarding their behavioral consequences (Zhao and Chubb, 2001; Brooks et al., 2002; Zimmer and Kovács, 2011a). Since there are both differences and similarities at the behavioral level, the degree to which the two phenomena share the same neuronal mechanisms is under debate. It is well-known that repetition of a particular stimulus usually lowers the firing rate of the responsive neurons, reduces the BOLD responses and the MEG/EEG signals (for reviews see Grill-Spector et al., 2006; Krekelberg et al., 2006). In the case of faces, various cortical areas and structures showed decreased activation in an fMRI adaptation paradigm (fMRI-A; Grill-Spector and Malach, 2001), such as the fusiform face area (FFA, Kanwisher et al., 1997), the occipital face area (OFA, Gauthier et al., 2000), the posterior superior temporal sulcus (pSTS, Puce et al., 1998), or the lateral occipital complex (LO or LOC, Malach et al., 1995). In electrophysiological studies, neural adaptation [or its clearest form: the so-called repetition suppression (RS)] paradigms provide a powerful technique to determine the functional properties of face-evoked ERP components and their relation to underlying neural processing modules (Eimer et al., 2011).

Regarding human face perception, electrophysiological studies have described a large, early positive (P1 or P100) and a negative (N170) wave over the occipito-temporal areas (Bentin et al., 1996; Eimer, 2000; Itier and Taylor, 2004a). Although several researchers noted that P100 also responds to faces when compared to other non-face objects (Itier and Taylor, 2004b; Herrmann et al., 2005a), most studies linked P100 rather to early pictorial encoding (Desjardins and Segalowitz, 2009). The N170 is typically regarded as a marker for the structural encoding of faces (Schweinberger, 2011). This component is larger in amplitude and shorter in latency to face stimuli than to non-face objects (Bentin et al., 1996; Rossion and Jacques, 2008) and has clear right hemisphere dominance (Bentin et al., 1996; Allison et al., 1999). The subsequent component of the ERP, the P2, is characterized by a positive-going deflection over the lateral occipito-temporal areas peaking at about 200–220 ms after stimulus onset. This component has been linked to the processing of facial relations between facial features in individual faces (Latinus and Taylor, 2006) and generally larger P2s were observed in tasks needing expertise (Stahl et al., 2008; Wiese et al., 2009). Altogether, this component might be involved in the deeper and more advanced analysis of faces when compared to earlier face-evoked components (Németh et al., 2014). In contrast to the N170, the second negative peak, the so-called N250 (or N250r), has consistently emerged as an electrophysiological correlate of face recognition (Schweinberger, 2011). This later negative component reaches its maximum between 230 and 330 ms after stimulus onset and is generated by the FFA (Eger et al., 2005).

The electrophysiological correlates of facial after-effects have been reported as early as 140–200 ms post-stimulus onset (in the time window of the N170 component). Following the initial study of Kovács et al. (2006), further works showed reduction of the N170 (or the M170 in magnetoencephalographic studies) when the adaptor image was a face when compared with noise stimuli or non-face objects (Harris and Nakayama, 2007, 2008; Kloth et al., 2010; Nemrodov and Itier, 2012). It is worth noting, however, that in most studies, this N170 adaptation to identity-congruent adaptors seems small or absent (Amihai et al., 2011). Even though the authors of previous face adaptation studies did not concentrate directly on the positive ERP components (P100 and P2), the increase of their amplitude after adaptation is evident in the grand average ERP figures of these studies (Kovács et al., 2007; Zimmer and Kovács, 2011b; Feuerriegel et al., 2015). These findings show that there are different modulating effects on the positive components (signal enhancement) when compared with the N170 as a consequence of adaptation, suggesting separate mechanisms that elicit these components and different roles that they play in face perception. It can also be hypothesized that the effect reflected on the positive components is linked to priming rather than to adaptation. The face-evoked ERP component that is more closely associated with face repetition priming is the N250 (N250r) component (Schweinberger et al., 2007b). Although a clear N250 amplitude increase was observed for repetitions of familiar faces across different images, this increase was larger for repetitions using the pixel-by-pixel same image when compared with different images of the same identity (Schweinberger et al., 2002a).

To the best of our knowledge, so far the only study testing priming and adaptation simultaneously within a single paradigm is a study by Walther et al. (2013). The authors presented pairs of stimuli, where the first stimulus was either a famous face (from three different identities) or a 50/50% morph between two famous faces and a Fourier phase-randomized image that served as control. The second, test stimulus was always a face from the morph continuum. In a 2AFC identity discrimination task the authors dissociated priming and adaptation effects based on the ambiguity of the test stimuli. They concluded that their results show that face-related adaptation and priming are both present in the same paradigm, but are never observed simultaneously for a given test stimulus. This indicates that exclusive mechanisms may underlie both adaptation and priming.

One of the important parameters affecting the mechanisms of adaptation is its timescale and this can be tested by either the manipulation of the exposition time of the adaptor or by using different adaptor-test image inter-stimulus intervals (ISIs). Determining how adaptation duration influences changes in neuronal response properties is central to the understanding whether the sensory system employs different strategies for adjusting its sensitivity on the different timescales (Patterson et al., 2013). From single cell recording results, we know that brief and prolonged adaptation can lead to qualitatively different changes in neural tuning. For example, adaptation takes place in motion direction selective neurons of macaque area MT after short but not after long exposure to their preferred motion direction (Priebe et al., 2002; Kohn and Movshon, 2003). In the case of the orientation selective neurons of V1, Patterson et al. (2013) found that adaptation with small gratings reduced responsivity and caused tuning shifts away from the adaptor grating. This effect became more pronounced with more prolonged adaptation durations. Brief and prolonged adaptation produced, however, indistinguishable effects on responsivity, but caused opposite shifts in tuning preferences in the case of large gratings. Regarding human face perception based on psychophysical data, it was shown that perceptual after-effects (namely the face identity after-effect) for simple visual attributes processed early in the cortical hierarchy increase logarithmically with adapting duration and decay exponentially with test duration (Leopold et al., 2005; Rhodes et al., 2007). In human electrophysiological [or magnetoencephalographic (MEG)] experiments where the adaptor and test stimuli are presented in rapid succession, strong N/M170 amplitude reductions are observed for every face (or face-part) adapted condition (Harris and Nakayama, 2007, 2008; Eimer et al., 2010; Nemrodov and Itier, 2011, 2012). In one of our previous studies, we manipulated the effect of adaptation duration and stimulus position together (Kovács et al., 2007). We have found that that for shorter adaptor durations (500 ms) the adaptation effect was entirely position independent, whereas for longer durations (5000 ms), the effect was partly position invariant but partly position dependent. As it has already been mentioned, other studies varied the ISI to test the timescale of adaptation. Daelli et al. (2010) presented prototypical adaptors (S1), followed by ambiguous target objects (S2), and found aftereffects when the ISI was short (50 ms) but priming effects when the ISI was long (3100 ms). An MEG study by Harris and Nakayama (2007) also investigated the effects elicited by different ISIs (100, 200, 300, and 600 ms) and found that the M170 response to the second face decreased in a linear manner with decreasing ISI. This shows that adaptation depends on the ISI, even though this effect is short-lived as suggested by the fact that for an ISI of 800 ms there was no difference between adapted and non-adapted conditions. In a recent work, Feuerriegel et al. (2015) measured the category-specificity of adaptation over a limited range of adaptor durations (200, 500, and 1000 ms) and for two short ISIs (200 and 500 ms). Their results indicated that, not surprisingly, at the level of N170 there was no category adaptation for faces. Face adaptors led to the smallest N170 amplitudes for both target faces and chairs after 500 ms adaptor duration.

In the present study, we systematically manipulated the duration of adaptation in the commonly applied range of 200–5000 ms with different facial adaptation conditions. In a 2AFC familiarity decision task we used three different face adaptors: different identity (DiffID), different image of the same identity (SameID), and identical image of the same person (repetition suppression, RS). A Fourier phase-randomized noise image served as control (No). By using these conditions we intended to separate three major steps of face processing. The GENERIC, face category specific processes were isolated by the comparison of noise adaptation (No) with the adaptation to a face that is unrelated to the test stimulus identity (DiffID). IDENTITY-SPECIFIC effects were tested by the DiffID vs. SameID comparison while IMAGE-SPECIFIC effects were measured by the SameID vs. RS comparison. We hypothesized that by varying the duration of adaptation time, we might dissociate generic-, identity-, and image-based information processing, reflected on the face-evoked ERP components.

## Materials and methods

### Participants

Sixteen naïve healthy-volunteers (15 right-handed and one ambidextrous, six females, mean age = 22.69 years, ± 4.83 years SD) participated in the study. They received partial course credits for their participation and gave signed, informed consent in accordance with the Ethical Committee of the Budapest University of Technology and Economics prior to testing. All participants had normal or corrected-to-normal visual acuity, no previous history of any neurological or ophthalmological diseases and were not under any medication. One additional participant was excluded from the behavioral and electrophysiological analysis (right-handed 22 years old male) due to an insufficient number of recognized familiar faces and a high number of recognized unfamiliar faces, which was evident in the post-testing questionnaire.

### Stimuli

Grayscale images of familiar and unfamiliar faces were used, with sixteen different identities (eight female) for each category. Familiar faces were digital images of famous persons, ranging from Hollywood celebrities to politicians that Hungarian students were likely to recognize, whereas unfamiliar faces were digital images of less-known persons that were unlikely to be recognized by the participants (such as Finnish politicians or actresses from Iceland). Two different images portraying the same person were used for each identity, corresponding to a number of 32 images in total. All images were downloaded from freely available websites and converted into grayscale (8 bit) using Adobe Photoshop CS3 Extended 10.0 (Adobe Systems Inc.). Images were then cropped to reveal only the contour of the face, including hair. Stimuli subtended 3.6 × 5° of visual angle. Since previous studies have shown that early ERP components, such as P100, are sensitive to luminance (Johannes et al., 1995) and that neural processes are sensitive to luminance histogram skewness (Olman et al., 2008), stimuli were equated in luminance and their histograms were matched using the *lummatch* and *histmatch* functions of MATLAB (Mathworks, Natick Massachusetts, USA) SHINE toolbox (Willenbockel et al., 2010).

Three different images were used as adaptors: an image that was identical to the test stimulus [repetition suppression (RS) condition], a different image depicting the same identity as the test stimulus [same identity (SameID) condition], and an image depicting a same gender different identity than the test stimulus [different identity (DiffID) condition]. For the control condition, sixteen Fourier phase-randomized images were created from the face images and used as adaptors [non-adapted (No) condition].

Stimuli were presented centrally on a uniform gray background. An LG Flatron W2600 HP monitor (resolution: 1920 × 1200 pixels, refresh rate: 60 Hz) was used for stimulus presentation, while viewing distance (57 cm) was maintained constant using a chinrest. Stimulus presentation was controlled using MATLAB 2008a Psychtoolbox 3.0.9 (Brainard, 1997; Pelli, 1997) and custom-made scripts.

### Procedure

**Figure 1:**
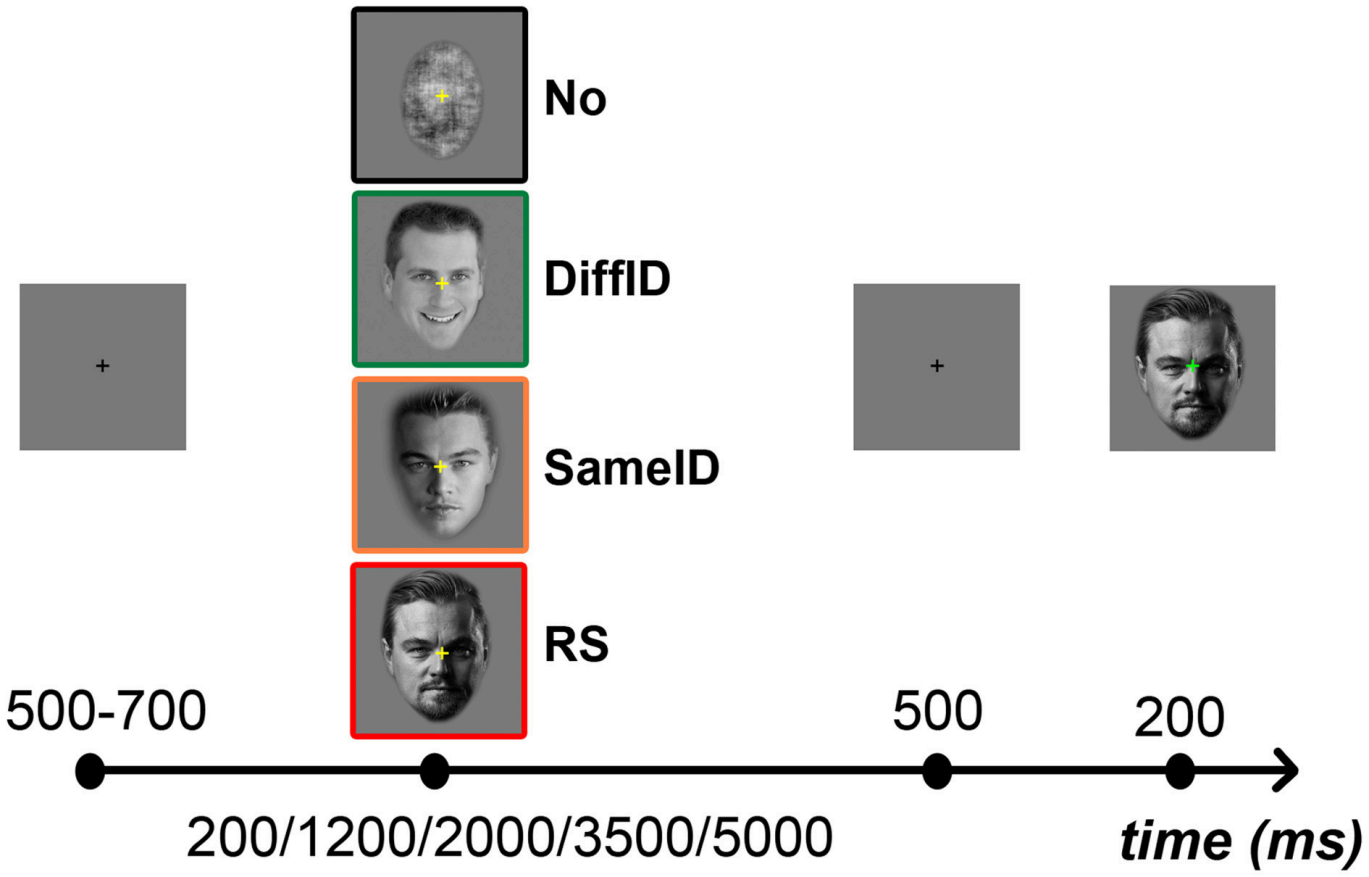
**Experimental protocol and illustration of the different types of adaptors**. In the beginning of each trial a fixation cross was presented in the center of the screen for a short interval ranging randomly between 500 and 700 ms, followed by one of the four adaptors (from top to bottom: No, signaled by a black frame; DiffID, signaled by green; SameID, signaled by orange; and RS, signaled by red) which was visible for either 200, 1200, 2000, 3500, or 5000 ms. This was followed by the presentation of a blank screen for 500 ms, and then the test face was displayed for 200 ms.

Subjects were instructed to perform a two-alternative forced-choice (2AFC) familiarity task for faces by pressing the key labeled “7” on a keyboard when the second, target face was perceived as familiar and the key labeled “8” when it was perceived as unfamiliar. A yellow fixation cross presented in the center of the screen indicated the presence of the target stimulus to the subject. Prior to the presentation of the target, a gray screen was presented for a random period of time between 500 and 700 ms, followed by the adaptor image. The duration of the adaptor image varied randomly between one of five values: 200, 1200, 2000, 3500, and 5000 ms. The adaptor was followed by a 500 ms gray screen after which the target stimulus appeared for 200 ms (Figure 1). To ensure that the subjects were also focusing on the adaptor image, a blue fixation cross directed their attention to the center of the screen during the presentation of the adaptor. Subjects were also instructed to refrain from movements during the experiment and from blinking during the presentation of the target stimulus. Each subject completed a total of 640 trials [2 (familiarity: familiar (F) vs. unfamiliar (UF)) × 4 (adaptor category: No, RS, SameID, DiffID) × 5 (adaptor duration: 200, 1200, 2000, 3500, 5000 ms) × 16 (identity)] with five breaks in between testing blocks. Adaptor categories and durations were intermixed and presented in random order. An experimental session lasted approximately 50 min.

At the end of the session participants had to answer a questionnaire that verified their familiarity with the identities used in the experiment. The questionnaire consisted of presenting each previously presented person and asking the participants to identify the familiar ones by naming them (or at least giving some correct information about them, such as occupation and nationality). In case the subject did not recognize one or more of the faces belonging to the familiar category or recognized faces belonging to the unfamiliar category, the trials containing those identities were removed from the statistical analysis for both behavioral and electrophysiological data. From the 32 faces on average 2.56 ± 2.25 faces were excluded from further analyses across participants.

### Behavioral data analysis

Accuracy and response times (RTs) were recorded during the experiment. In the case of accuracy, we calculated d′-values that were analyzed with a 4 × 5 repeated measures ANOVA with adaptor category (4; No, RS, SameID, DiffID) and adaptor duration (5; 200, 1200, 2000, 3500, 5000) as within-subject factors. RTs were analyzed with a 2 × 4 × 5 repeated measures ANOVA with familiarity (2; familiarity: F vs. UF), adaptor category (4; No, RS, SameID, DiffID) and adaptor duration (5; 200, 1200, 2000, 3500, 5000 ms) as within-subject factors. All analyses involved Greenhouse—Geisser adjusted degrees of freedom for correction for non-sphericity. *Post-hoc-t*-statistics were performed by Fisher's LSD tests.

### Electrophysiological recording and analysis

#### EEG acquisition and processing

Electroencephalography (EEG) data was recorded using a Brain-Amp (BrainProducts GmbH, Munich, Germany) amplifier from 60 Ag/AgCl scalp electrodes placed according to the international 10/10 electrode system (Chatrian et al., 1985) and mounted on an EC80 Easy Cap (Easycap, HerrschingBreitbrunn, Germany). Eye movements were recorded using two electrodes placed on the outer canthi of the eyes and one electrode placed on the infraorbital ridge of the right eye. All channels were referenced online to an average of the activity recorded at the two reference electrodes placed on the left and right earlobe and digitally transformed to a common averaged reference offline. The ground electrode was placed on the forehead and all input impedances were kept below 8 kΩ. EEG was digitized at 1000 Hz sampling rate (with an analog bandpass filter of 0.016–1000 Hz).

#### ERP data analysis

EEG data were analyzed using Brain Vision Analyzer 1.05.0002 (BrainProducts GmbH., Munich, Germany). After correcting ocular movement artifacts and digitally re-referencing to a common average, the EEG was segmented offline into 700 ms long epochs starting 100 ms prior to target stimulus onset and ending 600 ms after target stimulus onset. DC trend correction was applied, and a semi-automatic artifact rejection was implemented. Segments containing blinks, movement artifacts, and baseline drifts were rejected on the basis of visual inspection. After cleaning the EEG data, ~87% of the trials remained available for further analysis. It is worth noting, however, that the reason of removing a given segment in most cases (8% from the 13% removed trials) was that the familiarity of the given person was misjudged by the subject. EEG epochs were averaged separately for each condition and participant. Averages were band-pass filtered (Butterworth zero phase filter; 0.1–30 Hz; slope: 12 dB/oct) and baseline corrected using a 100 ms pre-stimulus baseline. The peak amplitude and latency of the individually averaged ERPs were extracted using a semiautomatic detection algorithm that identified the global maxima separately for each selected channels in a specific time window as follows. P100 was defined as a main positive deflection in the 80–130 ms time window, whereas the N170 was defined as a negative component in the 135–190 ms time interval. P2 was the second positive component in the 195–250 ms time window, while N250 was defined as the second negative component at around 230–330 ms.

P100 amplitude was measured over O1 (left hemisphere, LH) and O2 (right hemisphere, RH) electrode positions (Herrmann et al., 2005a,b). For the N170, the standard posterior-occipito-temporal sites that correspond to the P7, P9, and PO9 (LH) and P8, P10, and PO10 (RH) channels were used (Wong et al., 2009). P2 amplitude was measured over O1, PO3, and PO7 (LH) and O2, PO4, and PO8 (RH) (Wang et al., 2014), while the amplitude of the N250 component was measured over P7, P9, PO9, and TP9 (LH) and P8, P10, PO10, and TP10 (RH) (Schweinberger et al., 2002b). A Five-way repeated-measures ANOVA was conducted for the amplitude values of the pooled values of the relevant electrodes with familiarity (2; F vs. UF), adaptor category (4; No, RS, SameID, DiffID), adaptation duration (5; 200, 1200, 2000, 3500, 5000 ms), hemisphere (2; LH vs. RH), and electrode (3 for N170 and P2 or 4 for N250) as within-subject factors separately for each component. (It is worth noting that for the P100 component a Four-way repeated-measures ANOVA was applied since we used only two electrodes, one for the LH and RH recordings.) The Greenhouse—Geisser correction was applied to correct for violations of sphericity, while *Post-hoc-t*-statistics were computed using Fisher's LSD tests.

In addition, we defined three different types of effects: ADA_GENERIC_ as referred to the No vs. DiffID comparison; ADA_IDENTITY_ as referred to the SameID vs. DiffID comparison; and ADA_IMAGE_ as referred to the SameID vs. RS comparison. The magnitudes of electrophysiological adaptation effects were defined as the absolute value of the differences in amplitude that were calculated by subtracting the P100, N170, P2, and N250 amplitudes obtained during the relevant two adapted conditions. After all, we tested whether the different types of adaptations modify the ERP components differently. Therefore, a Four-way repeated-measures ANOVA was conducted on the electrophysiological adaptation indices with adaptation effect (3; ADA_GENERIC_, ADA_IDENTITY_, ADA_IMAGE_), ERP component (4; P100, N170, P2, N250), hemisphere (2; LH vs. RH), and duration (5; 200, 1200, 2000, 3500, 5000 ms) as within-subject factors. A Greenhouse—Geisser correction was applied to correct for violations of sphericity, while *Post-hoc-t*-statistics were computed using Fisher's LSD tests.

## Results

### Behavioral results

#### Accuracy

**Figure 2:**
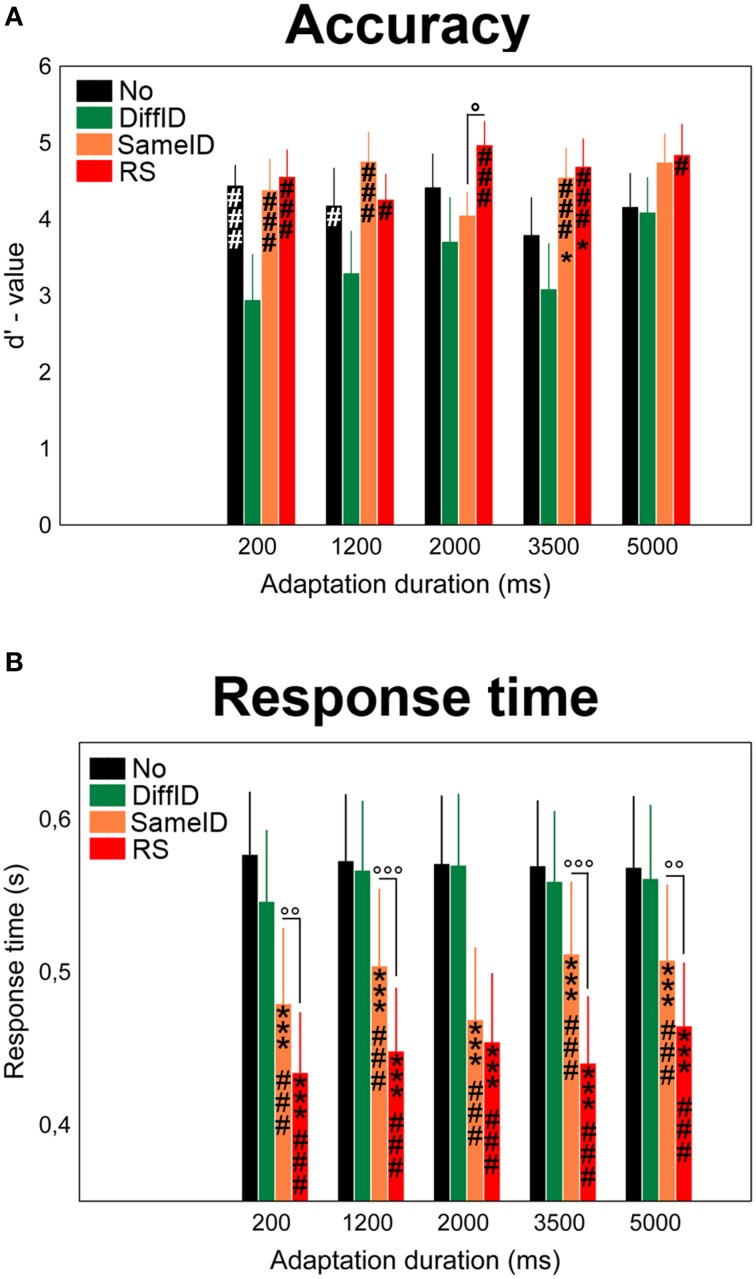
**Behavioral results**. Effects of varying adaptation durations on the accuracy **(A)** and response times **(B)** in the familiarity decision task. No (black), DiffID (green), SameID (orange), RS (red). “^*^” shows significant differences when compared with the control (No). “^#^” shows significant differences when compared with the DiffID condition. “°” shows significant differences between SameID and RS. One symbol indicates *p* < 0.05, two symbols indicate *p* < 0.01, and three symbols indicate *p* < 0.001.

We observed a main effect of adaptation CONDITION on the d′-values of the behavioral performance [*F*_(1.68, 25.22)_ = 7.56, *p* = 0.0003, $\eta_{p}^{2}=0.34$; Figure 2A]. *Post-hoc* comparisons suggested that it was due to a significantly worse performance (or according to the interpretation of the d′ analysis due to a significant lower sensitivity) in the case of DiffID when compared with other conditions (all *p*s < 0.009). Neither a main effect of DURATION [*F*_(4, 60)_ = 1.47, *p* = 0.22, n.s.] nor a significant CONDITION × DURATION interaction [*F*_(5.92, 88.74)_ = 1.53, *p* = 0.12, n.s.] were found.

#### Response times

A main effect of CONDITION was observed [*F*_(3, 45)_ = 61.54, *p* < 0.0001, $\eta_{p}^{2}=0.8$] in a step-by-step manner: the fastest decisions were observed for RS (all *p*s < 0.0001) followed by SameID (all *p*s < 0.0001), however, no significant difference was found between No and DiffID conditions (*p* = 0.3). No main effect of DURATION was found [*F*_(2.03, 30.5)_ = 1.19, *p* = 0.32], while adaptation duration modified the main effect of condition [significant CONDITION × DURATION interaction: *F*_(12, 180)_ = 2.76, *p* = 0.002, $\eta_{p}^{2}=0.16$]. We have observed longer RT-values in the case of 5000 ms adaptation duration when compared with 200 ms (*p* = 0.0033) and with 3500 ms (*p* = 0.019) in the RS condition. In the case of the SameID condition, on the one hand, faster decisions were observed for the shortest adaptation duration (200 ms) when compared with the other durations (all *p*s < 0.017) except for 2000 ms duration (*p* = 0.31). On the other hand, smaller RT-values were observed for 2000 ms duration when compared with longer durations (*p* < 0.0001 for both 2000 vs. 3500 and 2000 vs. 5000 comparisons). In the case of the DiffID condition, longer RTs were measured for 1200 and 2000 ms durations when compared with the shortest (200 ms) duration (*p* = 0.047 and *p* = 0.022, respectively). Faster decisions were observed for familiar faces when compared with unfamiliar ones, suggesting a significant priming effect [main effect of FAMILIARITY: *F*_(1, 15)_ = 5.27, *p* = 0.037, $\eta_{p}^{2}=0.26$; Figure 2B]. Although we have found that FAMILIARITY modifies the main effect of CONDITION [significant FAMILIARITY × CONDITION interaction: *F*_(2.19, 32.84)_ = 12.09, *p* < 0.0001, $\eta_{p}^{2}=0.45$], the only relevant difference was faster decisions for RS (*p* < 0.0001), SameID (*p* = *p* < 0.0001), and DiffID *p* = 0.043) but not for No (*p* = 0.21) for familiar faces when compared with unfamiliar ones.

### Electrophysiological results

In the subsequent ERP Results Section, we only focus on the three above-mentioned adaptation effects (GENERIC, IDENTITY-SPECIF, and IMAGE-SPECIFIC EFFECTS) and therefore we only discuss the main effects of CONDITION and DURATION, and the significant CONDITION × DURATION interactions in details. Any other significant main effects and interactions are presented in Tables 1, 2, respectively.

**Table 1:** **Other significant main effects**.

	**P100**	**N170**	**P2**	**N250**
	**Significant main effects**			
COND	*F*_(3, 45)_ = 9.83, *p* < 0.0001	*F*_(3, 45)_ = 47.12, *p* < 0.0001	*F*_(2.19, 32.8)_ = 12.63, *p* < 0.0001	*F*_(1.89, 28.33)_ = 11.94, *p* < 0.0001
	No < SameID *p* = 0.0002	No > SameID *p* < 0.0001	No < SameID *p* = 0.0003	No < RS *p* = 0.004
	No < RS *p* = 0.0001	No > RS *p* < 0.0001	No < RS *p* < 0.0001	DiffID < RS *p* < 0.0001
		DiffID > RS *p* = 0.001	Diff < RS *p* = 0.0003	
HEM	*F*_(1, 15)_ = 5.75, *p* = 0.03	*F*_(1, 15)_ = 7.58, *p* = 0.015	*F*_(1, 15)_ = 12.56, *p* = 0.003	*F*_(1, 15)_ = 4.46, *p* = 0.052
	RH dominance	RH dominance	RH dominance	strong tendency for LH dominance
FAM	n.s.	*F*_(1, 15)_ = 8.94, *p* = 0.009	n.s.	*F*_(1, 15)_ = 14.48, *p* = 0.0017
		larger for unfamiliar faces		larger for familiar faces

In case of negative components larger (>) means more negative.

**Table 2:** **Other significant interactions**.

	**P100**	**N170**	**P2**	**N250**
	**Significant interactions**			
COND × DUR	n.s.	*F*_(6.27, 93.99)_ = 3.33, *p* = 0.0002	*F*_(6.37, 95.58)_ = 2.4, *p* = 0.007	n.s.
		No vs. SameID all *p*s < 0.0001 for all durations	No vs. SameID all *p*s < 0.025 from 1200 ms duration	
		No vs. RS all *p*s < 0.012 for all durations	No vs. RS all *p*s < 0.0001 from 1200 ms duration	
		DiffID vs. RS all *p*s < 0.03 for durations longer than 1200 ms	DiffID vs. RS all *p*s < 0.04 from 1200 ms duration	
COND × FAMIL	*F*_(3, 45)_ = 3.44, *p* = 0.025	n.s.	n.s.	n.s.
COND × HEM	*F*_(3, 45)_ = 3.83, *p* = 0.016	*F*_(3, 45)_ = 5.45, *p* = 0.003	*F*_(3, 45)_ = 2.97, *p* = 0.042 stronger differences in RH	n.s.
COND × DUR × HEM	n.s.	n.s.	n.s.	*F*_(4.67, 70.1)_ = 2.15, *p* = 0.016
				No vs. SameID *p*s < 0.04 in case of longer durations (3500 and 5000 ms) in the RH (other *p*s > 0.2)
				No vs. RS all *p*s < 0.03 except for 1200 ms LH (*p* = 0.25)
				DiffID vs. RS all *p*s < 0.006

In case of negative components larger (>) means more negative.

#### P100

**Figure 3:**
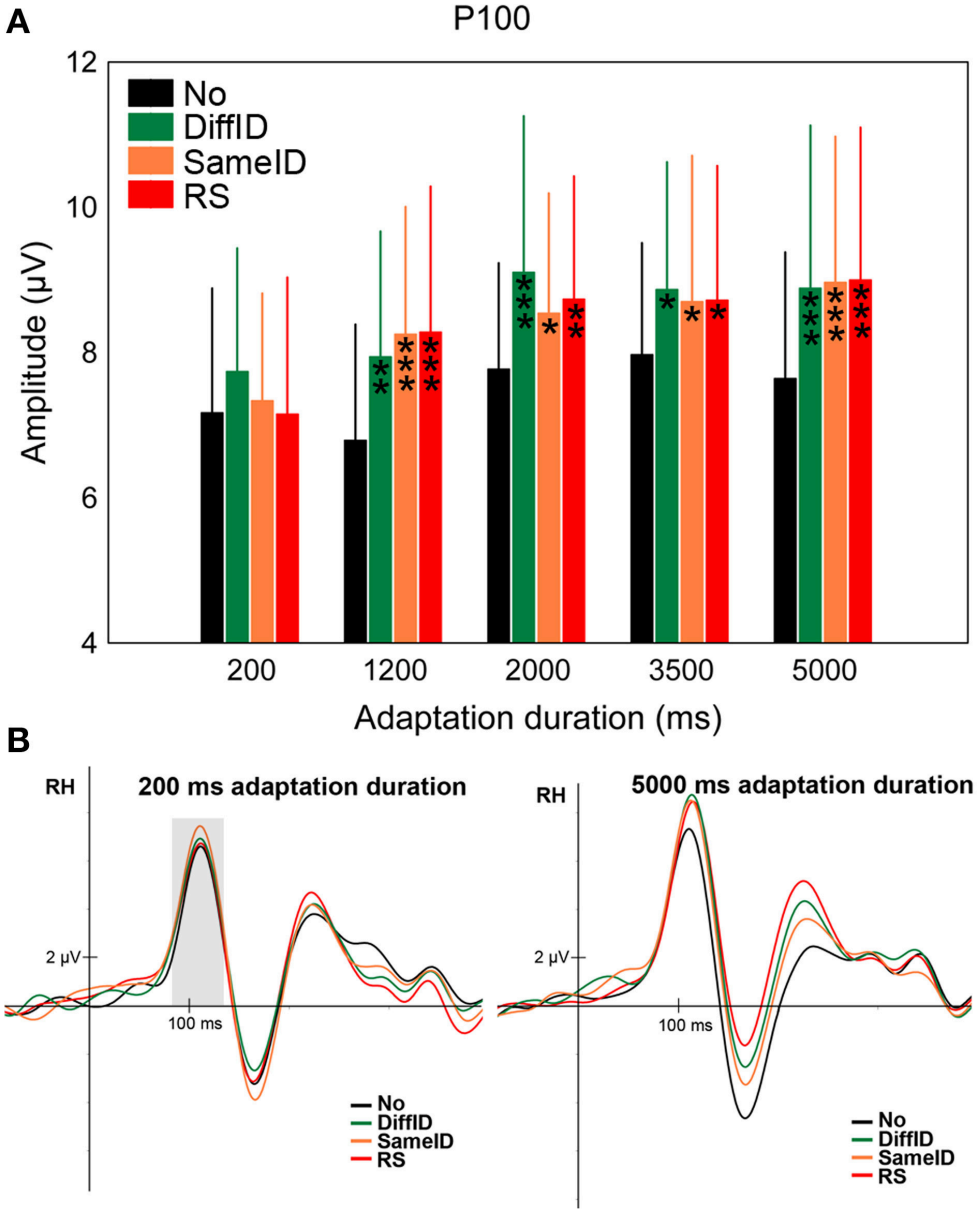
**P100**. **(A)** Average (±SE) amplitude values of the P100 component for the five adaptation durations (200, 1200, 2000, 3500, and 5000 ms), No, DiffID, SameID, and RS conditions. “^*^” shows significant differences when compared with the control (No). One symbol indicates *p* < 0.05, two symbols *p* < 0.01, and three symbols *p* < 0.001. **(B)** Sample grand average ERPs from O2 in No (black), DiffID (green), SameID (orange), and RS (red) conditions after short/rapid (200 ms) adaptation duration (lower left panel) and long (5000 ms) duration (lower right panel). Gray area marks the time-window where the component was analyzed on the respective RH electrode(s).

Significant GENERIC EFFECT was observed suggested by the larger P100 amplitude values in the DiffID condition when compared with the control (No) (main effect of CONDITION: *F*_(3, 45)_ = 9.83, *p* < 0.0001, $\eta_{p}^{2}=0.4$, *post-hoc p* < 0.0001; Figures 3A,B). Neither IDENTITY-SPECIFIC nor IMAGE-SPECIFIC EFFECTs were found (DiffID vs. SameID *p* = 0.49, SameID vs. RS *p* = 0.94). In the case of shorter adaptation durations (200 and 1200 ms), smaller P100s were measured when compared with longer adaptation durations [main effect of DURATION: *F*_(4, 60)_ = 12.09, *p* < 0.0001, $\eta_{p}^{2}=0.45$, *post-hoc* comparisons show all *p*s < 0.0028]. No other effects of duration were observed (all other *p*s > 0.71). Moreover, the main effect of condition was not modified by adaptation duration [non-significant CONDITION × DURATION interaction: *F*_(12, 180)_ = 1.34, *p* = 0.2]. Altogether, these results suggest that the P100 reflects only generic, face category specific adaptation processes and that this effect is independent of the adaptation time.

#### N170

**Figure 4:**
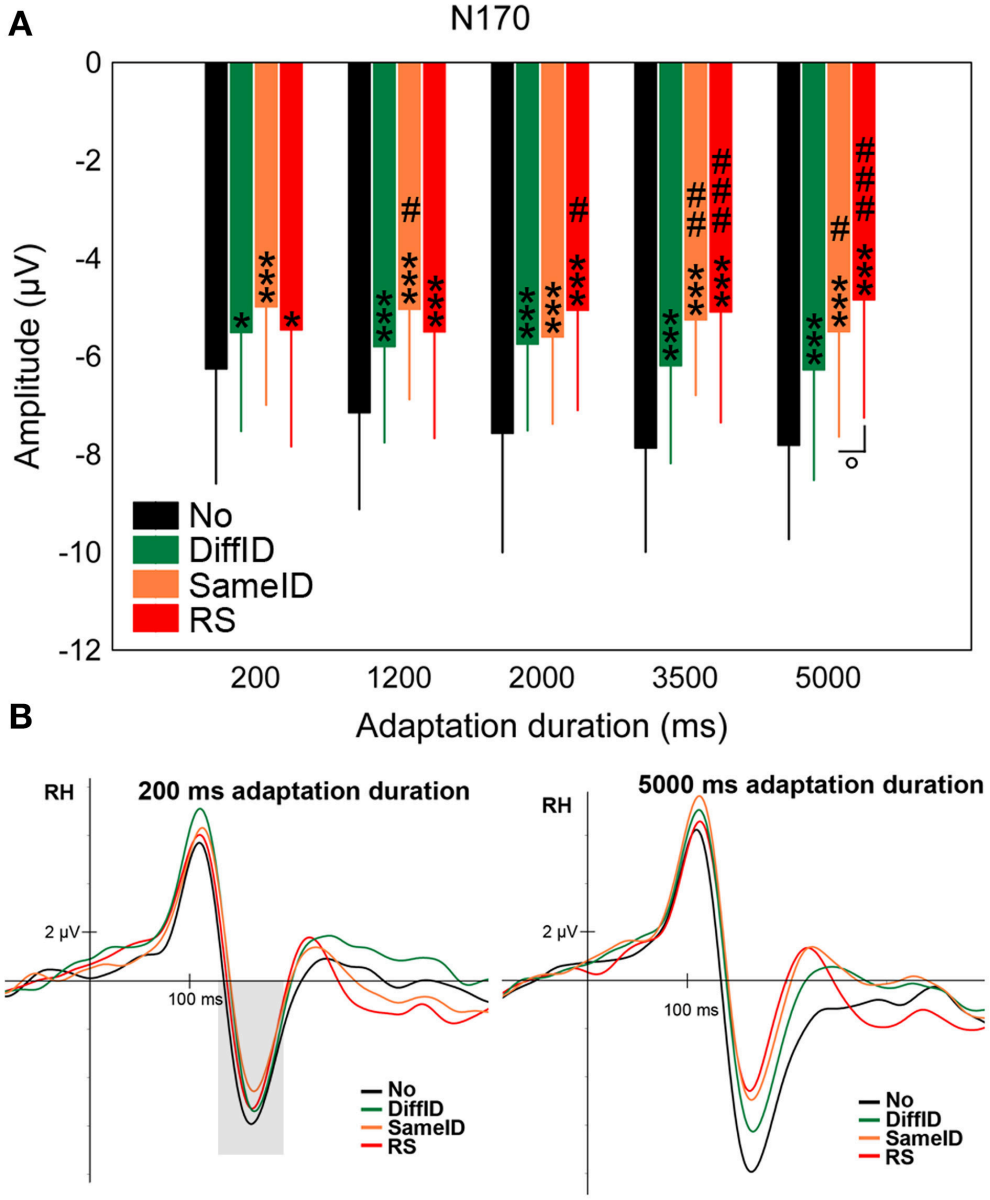
**N170**. **(A)** Average (±SE) amplitude values of the N170 component for the five adaptation durations (200, 1200, 2000, 3500, and 5000 ms), No, DiffID, SameID, and RS conditions. “^*^” shows significant differences when compared with the control (No). “^#^” shows significant differences when compared with the DiffID condition. “°” shows significant differences between SameID and RS. One symbol indicates *p* < 0.05, two symbols indicates *p* < 0.01, and three symbols indicates *p* < 0.001. **(B)** Sample grand average ERPs from RH (pooled from P8, P10, and PO10) in No (black), DiffID (green), SameID (orange), and RS (red) conditions after short/rapid (200 ms) adaptation duration (lower left panel) and long (5000 ms) duration (lower right panel). Gray area marks the time-window where the component was analyzed on the respective RH electrode(s).

The N170 showed a significant main effect of CONDITION [*F*_(3, 45)_ = 47.12, *p* < 0.0001, $\eta_{p}^{2}=0.76$] in a step-by-step manner (Figures 4A,B). The largest N170s were found in the case of the control (No) condition (all *p*s < 0.0001) followed by DiffID (all *p*s < 0.0035), however, no significant difference was observed between SameID and RS (*p* = 0.68) suggesting that at the level of N170 component, both GENERIC (*p* < 0.0001) and IDENTITY-SPECIFIC EFFECTs (*p* = 0.0035) can be observed but no IMAGE-SPECIFIC EFFECT (*p* = 0.68) is reflected on the component. Adaptation duration modified the main effect of condition [significant CONDITION × DURATION interaction: *F*_(6.27, 93.99)_ = 3.33, *p* = 0.0002, $\eta_{p}^{2}=0.18$]. In detail, GENERIC EFFECT was observed for all durations (all *p*s < 0.018), IDENTITY-SPECIFIC effect was found in the cases of 1200 and 3500 ms durations (*p* = 0.015 and 0.003, respectively), while IMAGE-SPECIFIC EFFECT was only observed in the case of the longest exposure time of the adaptor (5000 ms; *p* = 0.038) but not for the shorter ones (all *p*s > 0.09).

These results suggest that the N170 adaptation effects strongly depend on the adaptation duration: while for short adaptation durations the N170 reflects only generic, category specific effects, prolonging the adaptation duration leads to the emergence of identity and image specific adaptation effects as well.

#### P2

**Figure 5:**
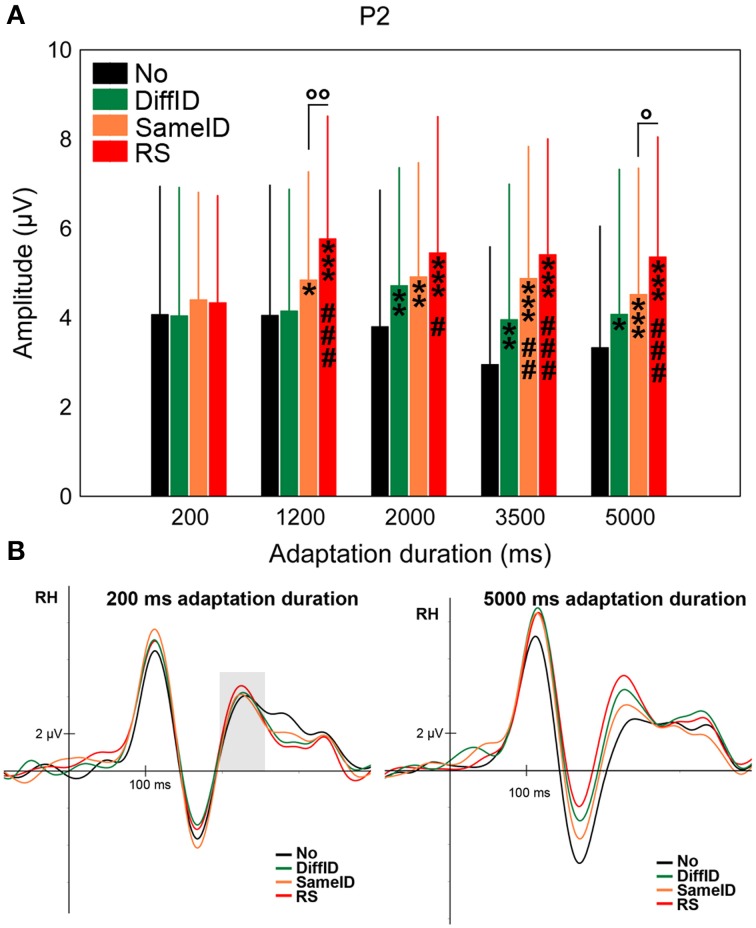
**P2. (A)** Average (±SE) amplitude values of the P2 component for the five adaptation durations (200, 1200, 2000, 3500, and 5000 ms), No, DiffID, SameID, and RS conditions. “^*^” shows significant differences when compared with the control (No). “^#^” shows significant differences when compared with the DiffID condition. “°” shows significant differences between SameID and RS. One symbol indicates *p* < 0.05, two symbols indicates *p* < 0.01, and three symbols indicates *p* < 0.001. **(B)** Sample grand average ERPs from RH (pooled from O2, PO4, and PO8) in No (black), DiffID (green), SameID (orange), and RS (red) conditions after short/rapid (200 ms) adaptation duration (lower left panel) and long (5000 ms) duration (lower right panel). Gray area marks the time-window where the component was analyzed on the respective RH electrode(s).

Although there was a strong tendency for both GENERIC EFFECT (namely larger P2s in the case of DiffID when compared with No, *p* = 0.054) and IMAGE-SPECIFIC EFFECT (larger P2s in the case of RS when compared with SameID, *p* = 0.053) and a somewhat weaker tendency for IDENTITY-SPECIFIC EFFECT (namely smaller P2s in the case of DiffID when compared with SameID, *p* = 0.065), neither of the comparisons reached the level of significance even though a significant main effect of CONDITION was observed [*F*_(2.19, 32.8)_ = 12.63, *p* < 0.0001, $\eta_{p}^{2}=0.46$] (Figures 5A,B). Another strong tendency was observed in the case of adaptation duration [main effect of DURATION: *F*_(4, 60)_ = 2.43, *p* = 0.057, $\eta_{p}^{2}=0.14$], suggesting slightly larger P2s in both 1200 and 2000 ms durations when compared with the shortest (200 ms) duration. The main effect of condition, however, was strongly modified by adaptation duration [significant CONDITION × DURATION interaction: *F*_(6.37, 95.58)_ = 2.4, *p* = 0.007, $\eta_{p}^{2}=0.14$]. In the case of the shortest adaptation duration, there were no significant effects reflected on the amplitude of the component (all *p*s > 0.3). In the case of longer durations (either 3500 or 5000 ms long exposures) all three effects were observed (GENERIC EFFECT: *p*s < 0.035, IDENTITY-SPECIFIC EFFECT *p*s < 0.009, and IMAGE-SPECIFIC EFFECT *p*s < 0.019).

#### N250

**Figure 6:**
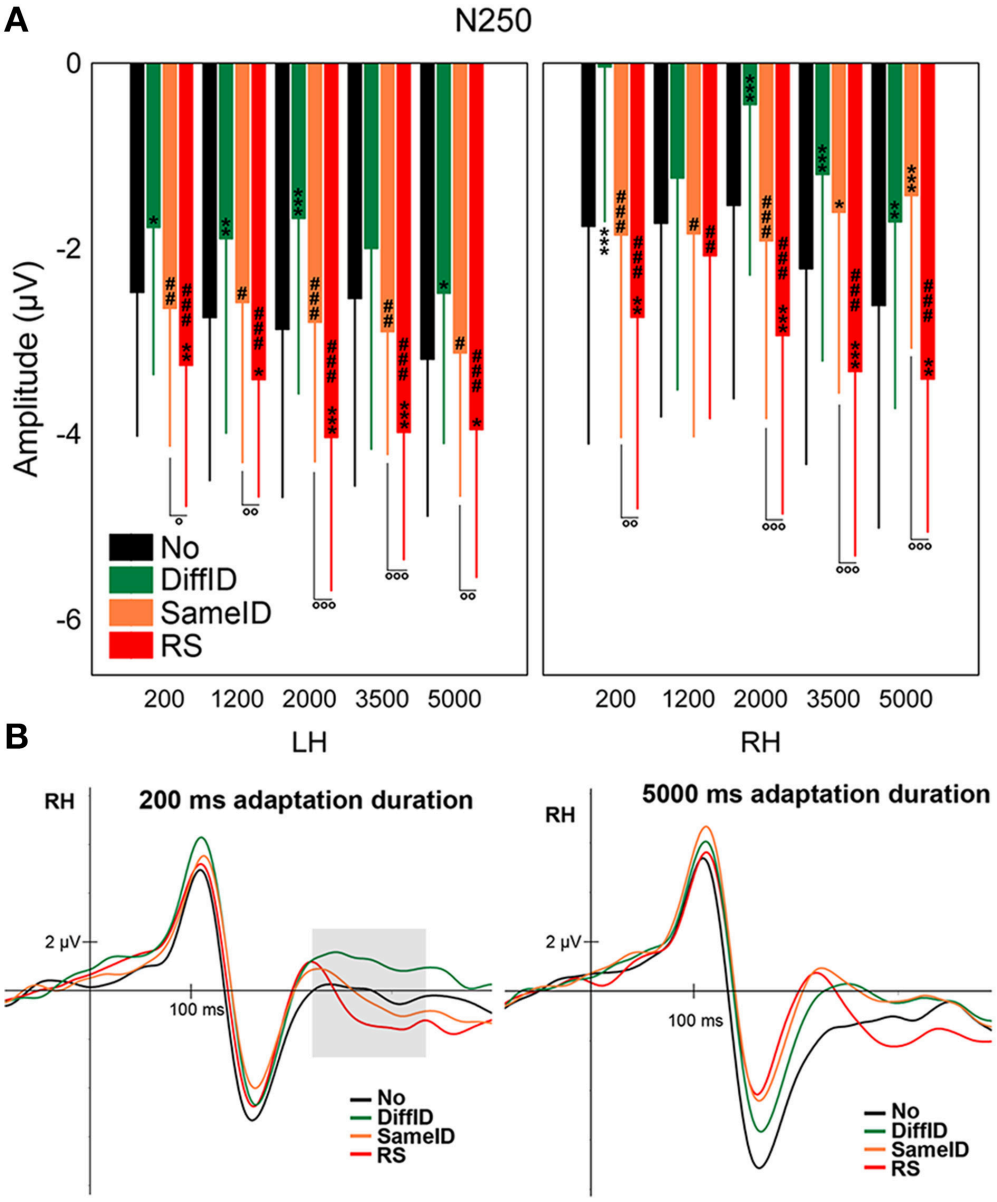
**N250**. **(A)** Average (±SE) amplitude values of the N250 component for the five adaptation durations (200, 1200, 2000, 3500, and 5000 ms), No, DiffID, SameID, and RS conditions. Upper left panel shows LH data while upper right panel displays RH data. “^*^” shows significant differences when compared the the control (No). “^#^” shows significant differences when compared with the DiffID condition. “°” shows significant differences between SameID and RS. One symbol indicates *p* < 0.05, two symbols indicates *p* < 0.01, and three symbols indicates *p* < 0.001. **(B)** Sample grand average ERPs from RH (pooled from P8, P10, PO10, and TP10) in No (black), DiffID (green), SameID (orange), and RS (red) conditions after short/rapid (200 ms) adaptation duration (lower left panel) and long (5000 ms) duration (lower right panel). Gray area marks the time-window where the component was analyzed on the respective RH electrode(s).

Supporting prior studies of N250r (Schweinberger et al., 2002a, 2004; Kaufmann et al., 2009), the most negative N250s were observed in the RS condition when compared with all other conditions [main effect of CONDITION: *F*_(1.89, 28.33)_ = 11.94, *p* < 0.0001, $\eta_{p}^{2}=0.44$, all *p*s < 0.0041]. We also observed less negative N250s for DiffID than for any other conditions (all *p*s < 0.012). N250s evoked by target faces were larger for the longest adaptation duration (5000 ms) when compared with the shorter durations [main effect of DURATION: *F*_(2.44, 36.66)_ = 3.02, *p* = 0.025, $\eta_{p}^{2}=0.17$, *post-hoc* comparisons: all *p*s < 0.035]. Although the statistical investigation of the CONDITION × DURATION interaction did not reach the level of significance [*F*_(12, 180)_ = 1.63, *p* = 0.09], significant three-way CONDITION × DURATION × HEMISPHERE interaction [*F*_(4.67, 70.1)_ = 2.15, *p* = 0.016, $\eta_{p}^{2}=0.13$] was found, suggesting that in the case of 1200 ms duration neither GENERIC (*p* = 0.11) nor IMAGE-SPECIFIC EFFECTs (*p* = 0.44) were observed (Figures 6A,B) over the right hemisphere. It was also the case for the two longest durations (3500 and 5000 ms) in the right hemisphere in the case of the IDENTITY-SPECIFIC EFFECT (all *p* = 0.18 for 3500 ms and *p* = 0.35 for 5000 ms). Since every other relevant comparisons were significantly different (all *p*s < 0.04) we can further emphasize the role of N250 in image and identity specific stimulus encoding.

By running an analysis based on different types of adaptation effects, we suggest that altogether larger adaptation effects were observed for GENERIC when compared with IDENTITY-SPECIFIC adaptation [main effect of ADAPTATION EFFECT: *F*_(2, 30)_ = 3.97, *p* = 0.03, $\eta_{p}^{2}=0.21$, *post-hoc* comparison: *p* = 0.01]. There was a weak tendency that ADA_IMAGE_ was slightly larger than ADA_IDENTITY_ (*p* = 0.066). Although no main effect of DURATION was observed [*F*_(4, 60)_ = 0.83, *p* = 0.51], the significant ADAPTATION EFFECT × DURATION interaction suggested that the ADA_GENERIC_ effects were the most pronounced for the 2000 and 3500 ms adaptation durations [*F*_(8, 120)_ = 2.21, *p* = 0.03, $\eta_{p}^{2}=0.13$, *post-hoc* comparison: all *p*s < 0.042]. Interestingly, ADA_GENERIC_ was the strongest in the case of 2000 ms duration, while at this duration the ADA_IDENTITY_ was the smallest one.

## Discussion

The goal of the present work was to study the behavioral and electrophysiological effects of systematically varying adaptation duration and whether this variation leads to a differentiation between the various stages of face processing, such as the generic category coding vs. processing of identity and image specific information. By using a large range of adaptation times we expected to find a differentiation between generic-, identity-, and image-specific processes.

The behavioral results indicated a better performance and faster decisions for the SameID and RS conditions when compared with the DiffID condition, corresponding to a strong priming effect in these cases (Ellis et al., 1987; Roberts and Bruce, 1989; Johnston and Barry, 2001). It is worth noting that the best performance and fastest decisions were observed in the case of RS when compared with the control (No) condition when the exposition time of the adaptor reached 1200 ms. In a recent study, Walther et al. (2013) found that the ambiguity of the stimuli regarding their classification plays an important role in differentiating between priming and adaptation-related after-effects with priming effects mostly found for unambiguous ones. This priming effect [namely in cases where the face adaptor image belonged to the same identity (SameID or RS) vs. to another identity (DiffID)] was to be expected, since the stimuli used in our experiment were unambiguous to the expected decision of the participants (as we used original photos without manipulating the familiarity information within the given face by morphing or other techniques that would have made the task more difficult). In the case of the speed of decision, we have found faster RTs for familiar faces, which is in line with earlier findings (Tong and Nakayama, 1999) that showed that familiar faces are processed faster than unfamiliar ones.

In the case of the P100 component, larger amplitudes were observed for all face-adapted conditions when compared with control (No) and the main effect of condition suggests a generic adaptation effect (ADA_GENERIC_) already at this level. This general signal enhancement is in line with our previous findings (Kovács et al., 2005, 2006, 2007; Zimmer and Kovács, 2011b), suggesting that there can be different modulating effects on the early P100 when compared to the later N170 as a consequence of adaptation, emphasizing separate mechanisms eliciting these components as well as their different roles in face perception.

All face adapted conditions caused a signal reduction of the N170 component when compared with noise adaptation (No). This general effect might be related to face detection and can be observed already after the shortest adaptation duration (200 ms). Recently, Feuerriegel et al. (2015) varied the exposition time of the adaptor stimulus systematically from 200 to 1000 ms and found no generic adaptation effect on the N170. These conflicting results can be explained by the fact that Feuerriegel et al. (2015) compared faces against another object category (chairs) while in the present study a uniform noise pattern was used as control condition. From the results of rapid adaptation studies where the authors tested the category-specificity of the adaptation effect (comparing ERPs evoked by face-adapted faces vs. non-face object adapted faces), we know that 200 ms duration is not enough to produce category-specific adaptation effects within the time-window of the N170 component (Nemrodov and Itier, 2012). It is worth noting, however, that Tian et al. (2015) have emphasized that the length of the ISI plays also an important role in the strength of the adaptation effect. In a rapid adaptation paradigm the authors have found both within-category (face adapted faces) and cross-category (house adapted faces) adaptation effects but only in the case of relatively shorter (~450 ms) ISI while in the case of longer ISI (~850 ms) only face-sensitive adaptation effects were observed. Therefore, it is possible that (at least at the shortest adaptation times) the N170 is also sensitive to the presentation of an object shape or Gestalt, a hypothesis requiring further tests. Moreover, since both SameID and RS adaptors are related to the test stimulus, it is possible that the bilateral reduction of the N170 component is also related to the processing of identity, a conclusion also supported by earlier findings (Jacques and Rossion, 2006; Jacques et al., 2007; Caharel et al., 2009). It is worth noting, however, that this additional signal reduction when compared to the generic effect is present only for the longest adaptation duration (5000 ms). This result also supports the claims of Nemrodov and Itier (2012) regarding the validity of rapid adaptation paradigms in testing category specific processes.

Regarding the P2 component, in the case of the shortest duration, there was no significant difference between conditions. However, as adaptation duration reached 1200 ms a step-by-step differentiation was found among No, DiffID, SameID, and RS conditions in an ascending manner for both hemispheres. This means that for adaptation durations longer than ~1000 ms the P2 component reflected generic, identity-specific and image-specific effects as well. Whereas the P100 and N170 components are clearly linked to special and well-defined stages of face processing, this does not seem to hold for P2. Some studies linked this component to task difficulty (Philiastides et al., 2006) while others emphasized the role of P2 in face-related tasks for which we have expertise (such as in case of own race effect, Stahl et al., 2008). P2 enhancement was also found recently investigating the effect of added noise (Bankó et al., 2011, 2013; Németh et al., 2014). Even though the LO/LOC is believed to be an object-selective area, some studies have found that its caudal part is also responsive to faces. Indeed, Nagy et al. (2012) and Hermann et al. (2015) have found bidirectional connections between the LO and the face-selective OFA-FFA complex, with faces modulating the LO-FFA connection and objects modulating the LO-OFA connection. Moreover, it is known that the neural generator of both P100 and P2 is the LOC (for review, see Schendan and Lucia, 2010). Therefore, one would expect a similar behavior of these two components—namely a large and pronounced enhancement for repeated stimulus presentations (RS). Since the largest alteration was measured between RS and control (No) and considering that LOC is involved in the generation of this component one could interpret this signal enlargement as a re-entrant loop from the OFA-FFA complex to the LOC. It is worth noting, however, that there are studies emphasizing other brain regions that can be involved in the generation of the early visual components, such as P100. Studies using less complex visual stimuli (isoluminant checkerboard stimuli) differentiated an early and a late phase of the P100 component that were localized to sources in dorsal extrastriate cortex of the middle occipital gyrus, and to sources in the ventral extrastriate cortex of the fusiform gyrus, respectively (Di Russo et al., 2001, 2005). Other source localization studies that used human face stimuli also have mentioned the fusiform gyrus as the neural generator of the P100 component (Herrmann et al., 2005b).

Regarding N250, not surprisingly, familiar faces evoked larger N250s, supporting prior results (Begleiter et al., 1995; Schweinberger et al., 1995; Pfüzte et al., 2002). At the level of this component generic-, identity-, and image-specific effects were found equally for the shortest and longest durations. These findings show that all observed adaptation effects (generic, identity-specific, and image-specific) happen in parallel and not independent from one another in this time-window.

**Figure 7:**
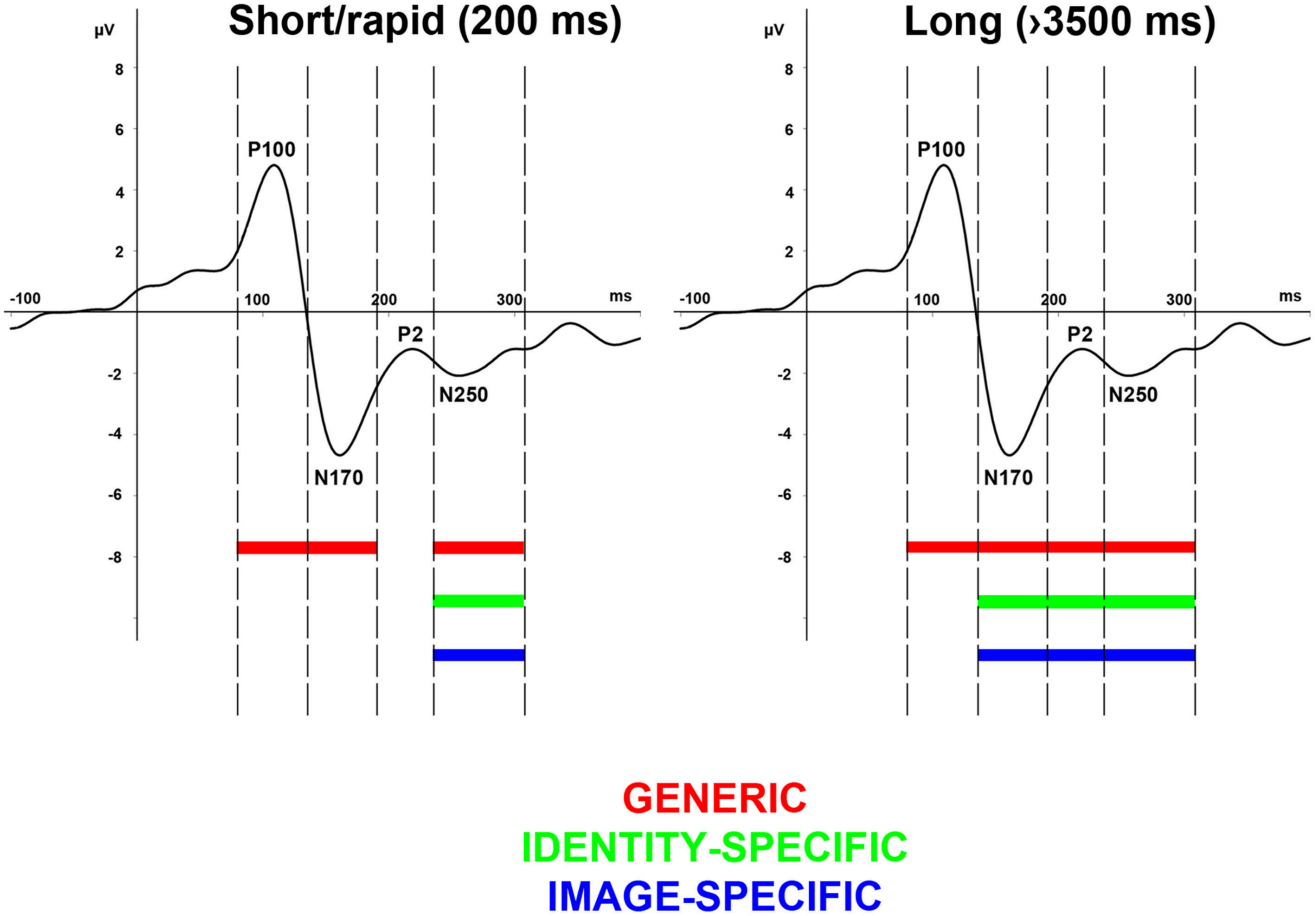
**Effect of adaptation duration on generic-, image-specific, and person-specific information**. A clear dissociation can be observed among the generic (red), image-specific (green), and identity-specific (blue) processing steps, reflected on the alterations of the earlier face-related ERP components but only in case of longer adaptation durations (left panel) and not in case of rapid adaptation (left panel).

Our results show that on the one hand, the observed face-evoked ERP components reflect generic, identity-specific, and image-specific adaptation effects that are modulated by adaptation duration in a way that in the case of the shortest duration (commonly applied in rapid adaptation paradigms) only the earlier P100 and N170 components reflect generic adaptation effects (for summary see our model on Figure 7). On the other hand generic, identity-, and image-specific effects can equally be observed on the amplitude modulation of the P2 and N250 components. In the case of longer durations however, generic effects have already been measurable from the earliest face-evoked responses onwards while identity-, and image-specific effects are present beyond the N170 component.

In conclusion, the current study is the first to investigate the relationship between systematically varying the adaptor duration in a broad range and the behavioral and electrophysiological responses elicited by the test stimuli after adapting different processing stages. Our results suggest that after long-term adaptation generic, identity-specific, and image-specific effects are reflected equally and earlier on the ERP components when compared to short-term adaptation conditions. In the case of rapid adaptation, these effects are delayed and can be observed only on the N250 component. Altogether we can summarize the consequences of our findings as follows: (1) While prior results rather suggested a sequential processing of faces, starting with a generic face categorization at about 150 ms (within the time-window of the N170 component), and identification/recognition being associated with three later components at around 250 ms (within the time-window of the N250 component), ours is the first study that clearly shows that with long-term adaptation these processes can all be observed from early components onwards and they are parallel to each other. (2) Our results suggest that in order to see the identity-specific processing steps completely, one needs to apply several seconds-long adaptation. (3) Our results also support those single-cell recording studies that suggest that short- and long-term adaptation have entirely different neural mechanisms (Priebe et al., 2002; Kohn and Movshon, 2003; Patterson et al., 2013).

## Author contributions

Designed the experiment: MZ, KN, GK; data acquisition: MZ, AZ, KN; data analyses: MZ, AZ, GK; interpretation of the data: MZ, AZ, GK; provided materials: MZ, AZ, KN, GK; wrote the article: MZ, AZ, GK; proofed/revised the article: MZ, AZ, KN, GK.

## Funding

This work was supported by a Deutsche Forschungsgemeinschaft Grant (KO 3918/1-2; 2-1).

### Conflict of interest statement

The authors declare that the research was conducted in the absence of any commercial or financial relationships that could be construed as a potential conflict of interest.
